# ‘It gives you nothing but it takes away everything’: Photo ethnography as a lens onto the experiences of people who inject drugs in Puerto Rico

**DOI:** 10.17157/mat.5.5.622

**Published:** 2018-12-19

**Authors:** Roberto Abadie, Colleen Syron, Carmen Ana Devila, Angelica Rivera-Villegas

**Affiliations:** Department at the University of Nebraska-Lincoln. Drawing on fieldwork conducted in Latin America, Puerto Rico, and New York City; University of Nebraska-Lincoln. She has worked for more than twenty years in branding, advertising, and storytelling.; University of Nebraska-Lincoln. She has conducted research on health disparities both in New York City and Puerto Rico.; University of Nebraska-Lincoln. She has conducted research about people who inject drugs and other vulnerable populations in Puerto Rico.

This essay offers insight into the material, social, and emotional worlds of intravenous drug use in rural Puerto Rico. Puerto Rico hosts one of the highest incidences of HIV infection in the United States (Centers for Disease Control and Prevention 2010), largely attributable to high rates of injection drug use. Visual ethnography is a powerful tool for exploring HIV risk behaviors and the materiality of injection drug use (Padilla et al. 2018; Moletsane et al. 2007), as well as the social and emotional worlds of people who use drugs (Clark-Ibáñez 2004; Madrigal et al. 2014). In contrast to photovoice, a method that can presume the empowerment of participants through the creation of images and that may require them to learn how to take high-quality pictures (Luttrell 2010), visual ethnography both involves participants in the creation of images to represent aspects of material, social, and emotional life and employs fieldwork methods to provide context for the meanings elicited by the pictures.

As part of a larger, two-year study of people who inject drugs in rural Puerto Rico (Abadie et al. 2016), we conducted extensive fieldwork to document the material practices that support intravenous drug use, shadowing participants as they hustled for drug money and partnered with other users to acquire and use drugs, and visiting shooting galleries and other settings where drugs are used. To further explore the material, social, and emotional dimensions of substance use we provided eighteen participants with disposable cameras and prompted them to take pictures of things that made them happy or sad, things they liked to do, and things they needed to inject drugs. In addition, all participants were invited to our office, where we took their portraits while they held hand-written signs with messages of their choosing. Participants were interviewed about the content of their photographs as well as the messages they chose for their portraits.

Incorporating this visual methodology allowed participants to express emotions and perspectives about their drug use that we had not been able to capture through fieldwork and more structured methods, such as surveys and interviews. It also provided valuable insights into the social, spatial, material, and emotional worlds of people who inject drugs in rural Puerto Rico. However, while this method opened a window onto the worlds of our study population, it also raised important ethical questions and had limitations. One concern was that participants might place themselves at risk when photographing their everyday drug use (including buying) practices, in order to receive the US$20 in financial compensation that was given to participants as part of the visual component of our study. We explicitly asked participants not to take pictures in places that involved even minimal risk (such as pictures of themselves or others buying or selling drugs), but we were also aware that nobody knows the unspoken laws that govern street drug culture in our setting better than the people who use those drugs. The locations that participants did choose to photograph revealed important zones of safety in their everyday lives. Despite the oftentimes dangerous daily hustle to buy drugs, many participants were able to retreat to the privacy of their homes, which were far removed from the harsh materiality of drug use in shooting galleries and other drug-dealing spots. These safe retreats, however, did not protect them from the continuous sense of loss embedded in drug consumption in the margins.

## *El chutin* (The shooting gallery)

Depending on injection frequency (half of the participants in our larger study injected four or more times per day), participants needed anywhere from US$11 to US$100 – or even more –each day in order to purchase small bags of cocaine and heroin. Of the participants in the larger study, 87 percent were unemployed and resorted to various hustles to afford their drug of choice, including begging at the entrances of high traffic stores, such as pharmacies, bakeries, discount stores, and banks; washing cars; bagging or carrying groceries at the supermarket; and monitoring parking lots. Drugs were usually acquired and used individually, but participants sometimes pooled resources to acquire and use drugs together.

Participants usually carried on them the syringes, bottled water, and cookers they needed to prepare drugs, sometimes in highly personalized bags.

For those who did not have homes to retreat to, a shooting gallery, colloquially known in Puerto Rico as ‘*el chutin*’ (a Spanglish deformation of ‘shooting’), was a preferred drug-using location. These places were also used by those who wanted to consume drugs immediately after purchase. Most shooting galleries lacked running water and electricity, and were almost devoid of comfort; sometimes they contained only a broken chair or sofa. While some such galleries in larger cities like San Juan or Caguas have a ‘manager’ who keeps the site relatively clean – providing users with clean needles for a fee and picking up used needles from the floor, for example – shooting galleries in rural areas have too little traffic to make this kind of arrangement profitable. Cleanliness in these places varies widely, which means some have piles of rubbish and used needles on the floor.

## Loss

‘Look at me’, writes la Chillin in her studio portrait. Her life circumstances were clearly visible to everyone, including her. ‘My body speaks’, she said, inviting us to ‘look at [her] scars’, the dime-sized holes in her arms the result of years injecting crack cocaine.

Many of the participants in our photo ethnography seemed to agree with Orvi, who lost a leg because of an untreated infected abscess: ‘The drug is not easy: it gives you nothing but it takes away everything’. For participants, loss included the loss of physical integrity and health but also the separation from husbands and wives, the taking away of sons and daughters by social services, and the disappearance of homes, cars, careers, hopes, and aspirations.

## Home sweet home

A number of participants took pictures of shooting galleries, particularly if they were there alone or with trusted drug-using partners. It is important to note that some drug locations were off-limits for photography. In *puntos* (as drug-selling spots are known in Puerto Rico), even mobile phones must be concealed in order to make clear that photographs are not being taken that could incriminate or expose drug dealers to retaliation.

While participants felt it was important to document – when possible – those places that they connected with the hustle to procure drugs, they also wanted to emphasize the importance of the homes they had been able to create, which were generally far removed from the grind of the street. While one in three participants in the larger study had been homeless during the past year, most of those who participated in the study’s photography activities were not homeless. They generally identified their houses as safe spaces, where they could escape from streets and enjoy their high while cleaning, playing with pets – from dogs and cats to roosters – or watching TV. Many participants also took care of plants or tended gardens while at home.

## Conclusion

Participants’ engagement with the images they produced offered them the opportunity to talk not only about issues related to the materiality of drug use but also the emotions bound up in their drug use, particularly issues of loss and social identity. These emotional worlds were less accessible through the other methods we employed. Even for those who could enjoy using drugs in the safety of their own homes, a sense of loss – of having lost material possessions but also of abandoned dreams and desires – did not leave them. Combining in-depth fieldwork and visual methods is a valuable means of tapping into not just the material, social, and spatial worlds of marginalized groups, such as people who inject drugs, but also their emotional worlds.

## Figures and Tables

**Figure 1.**
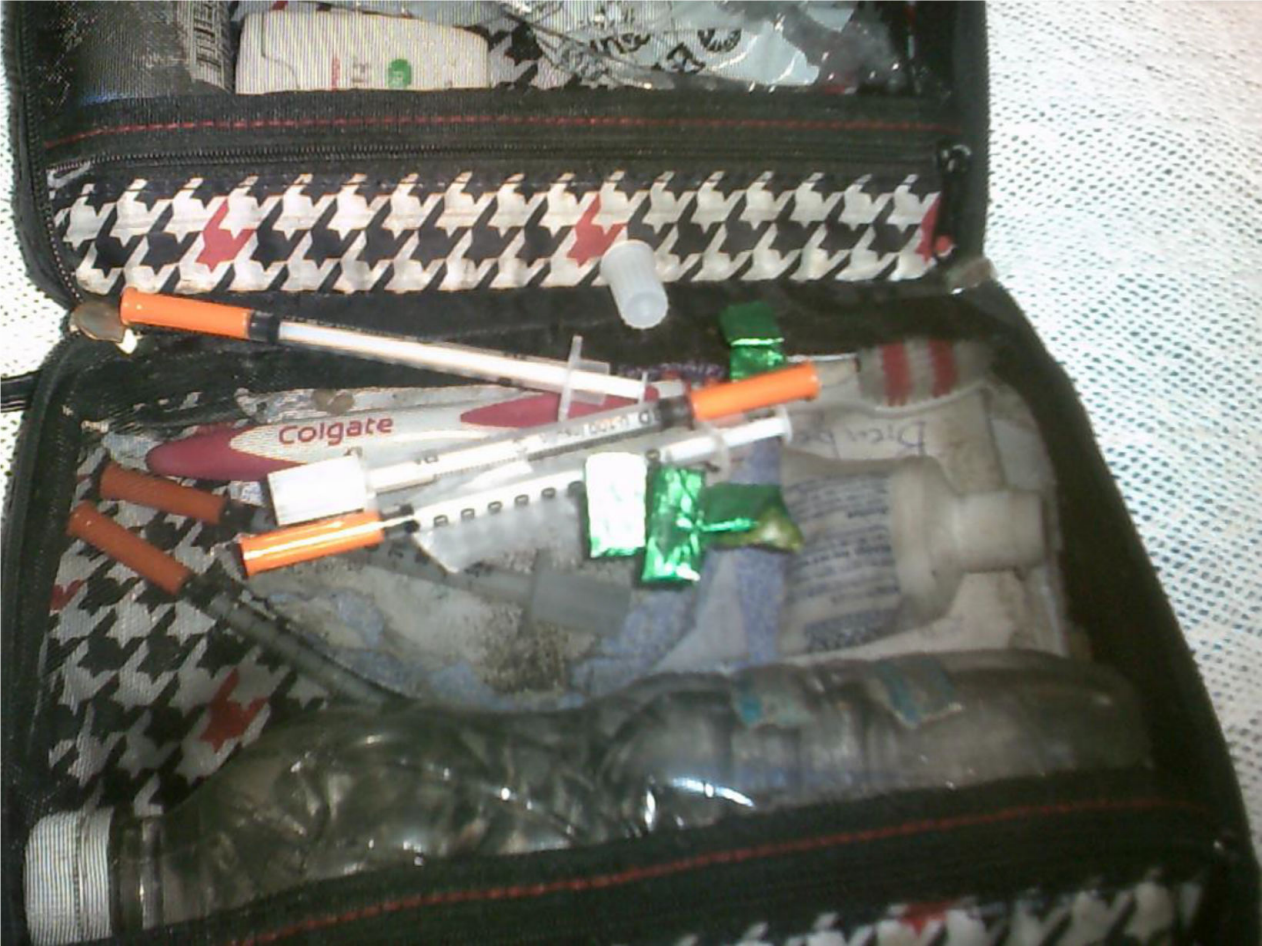
Toiletry bag with injection ‘works’

**Figure 2.**
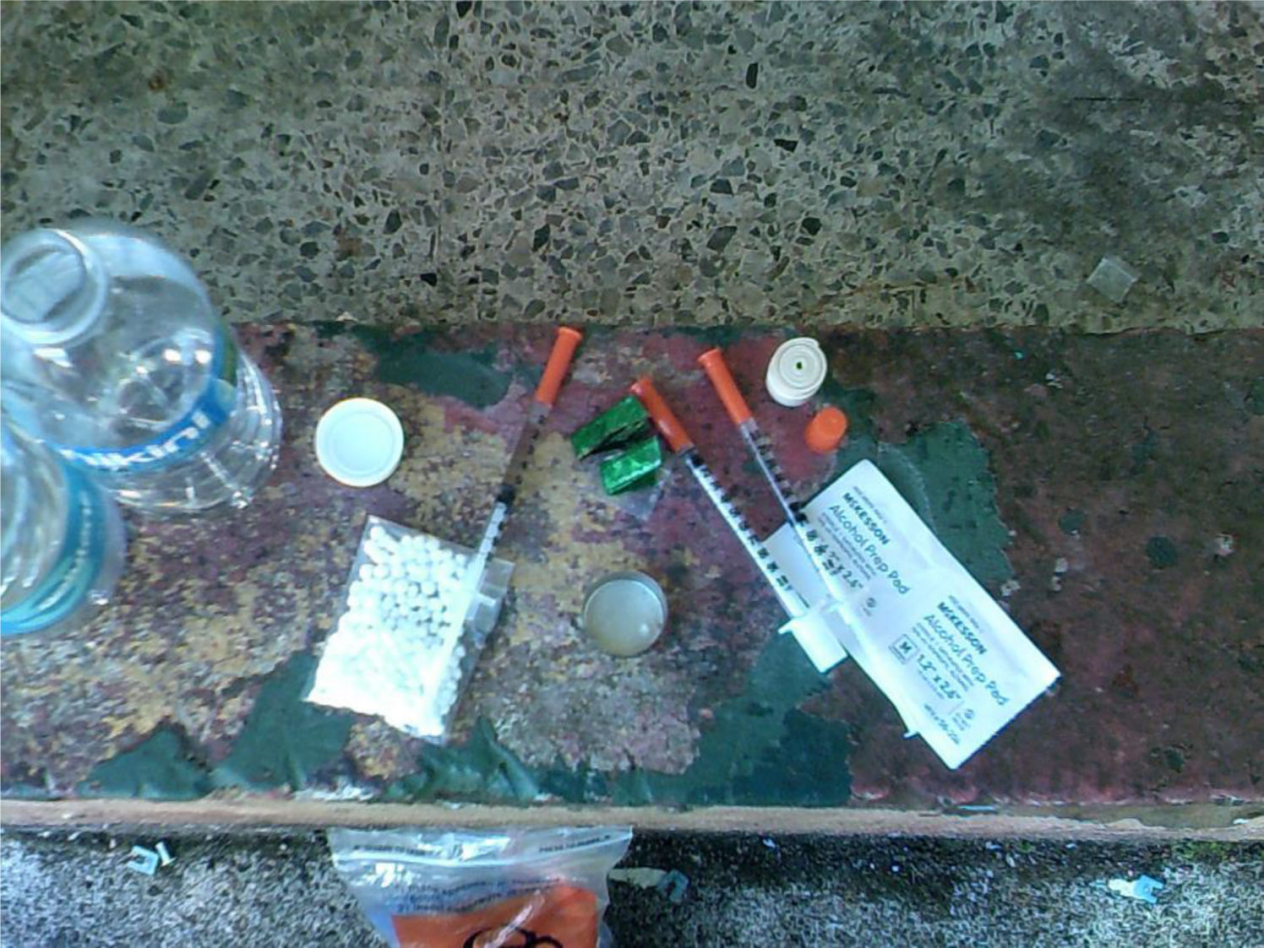
Syringe, cooker, and water on top of an improvised table at a shooting gallery

**Figure 3.**
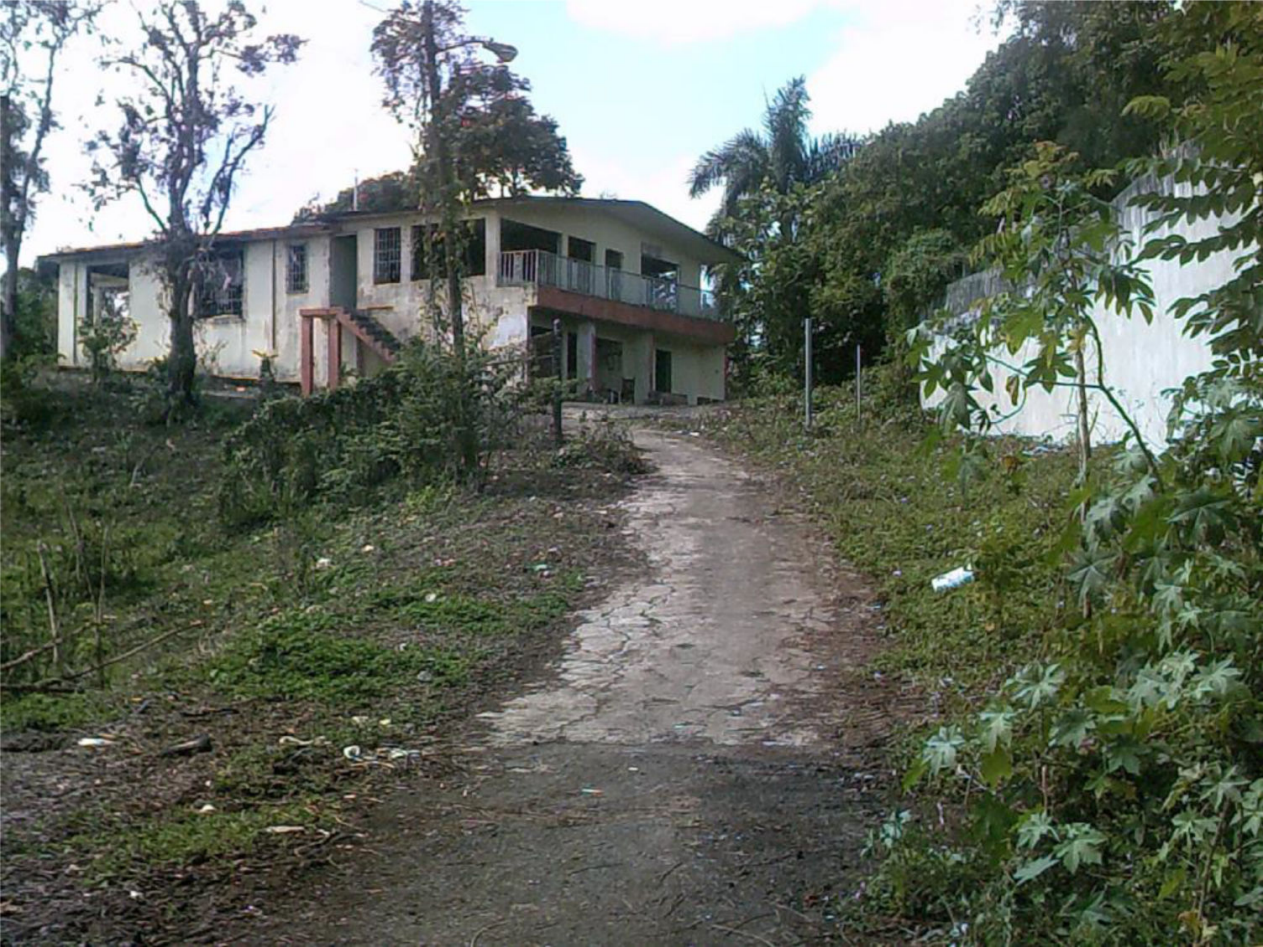
Shooting gallery in Cidra with a panoramic view

**Figure 4.**
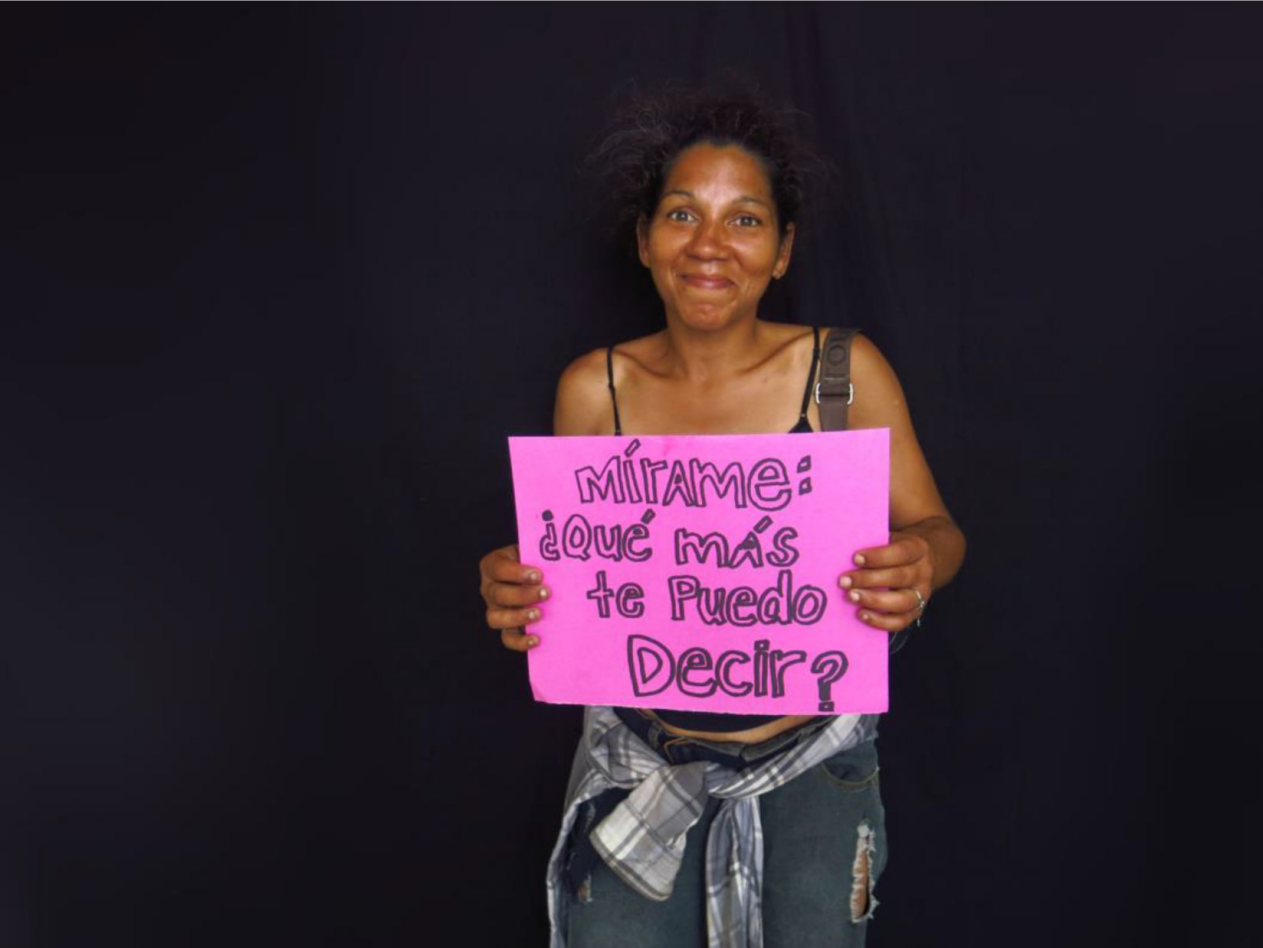
La Chillin portrait

**Figure 5.**
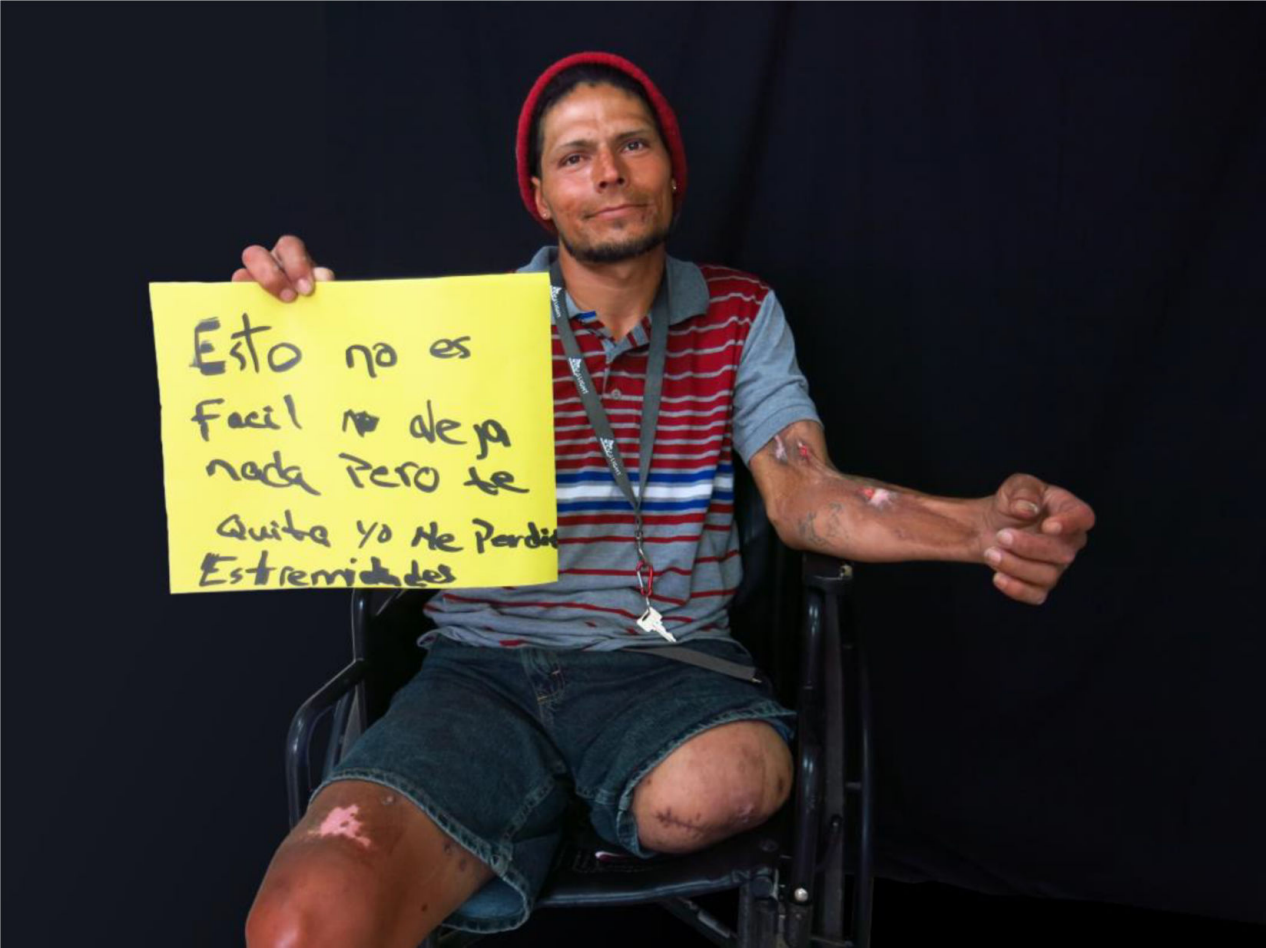
Orvi portrait

**Figure 6.**
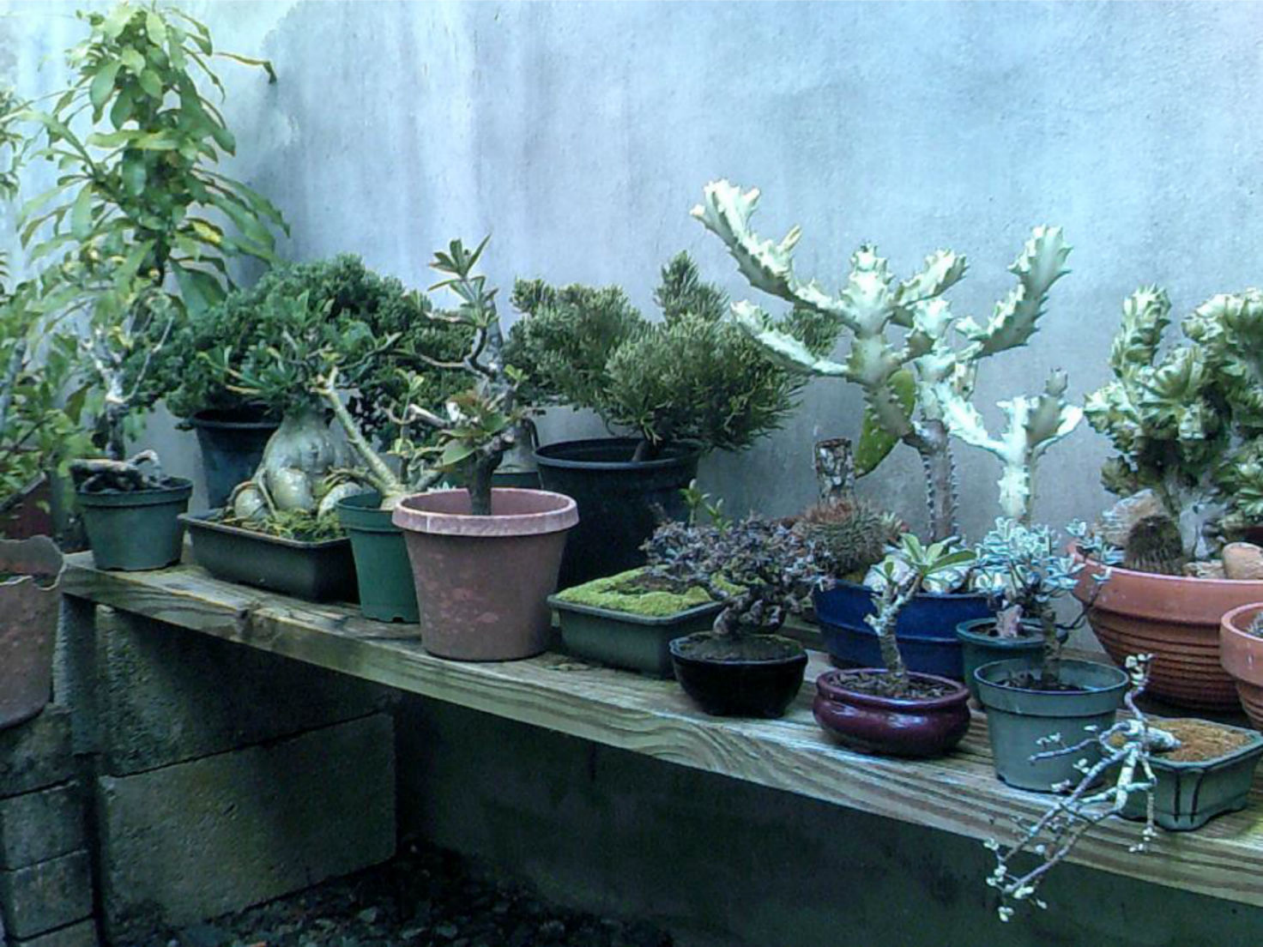
Bonsai

**Figure 7.**
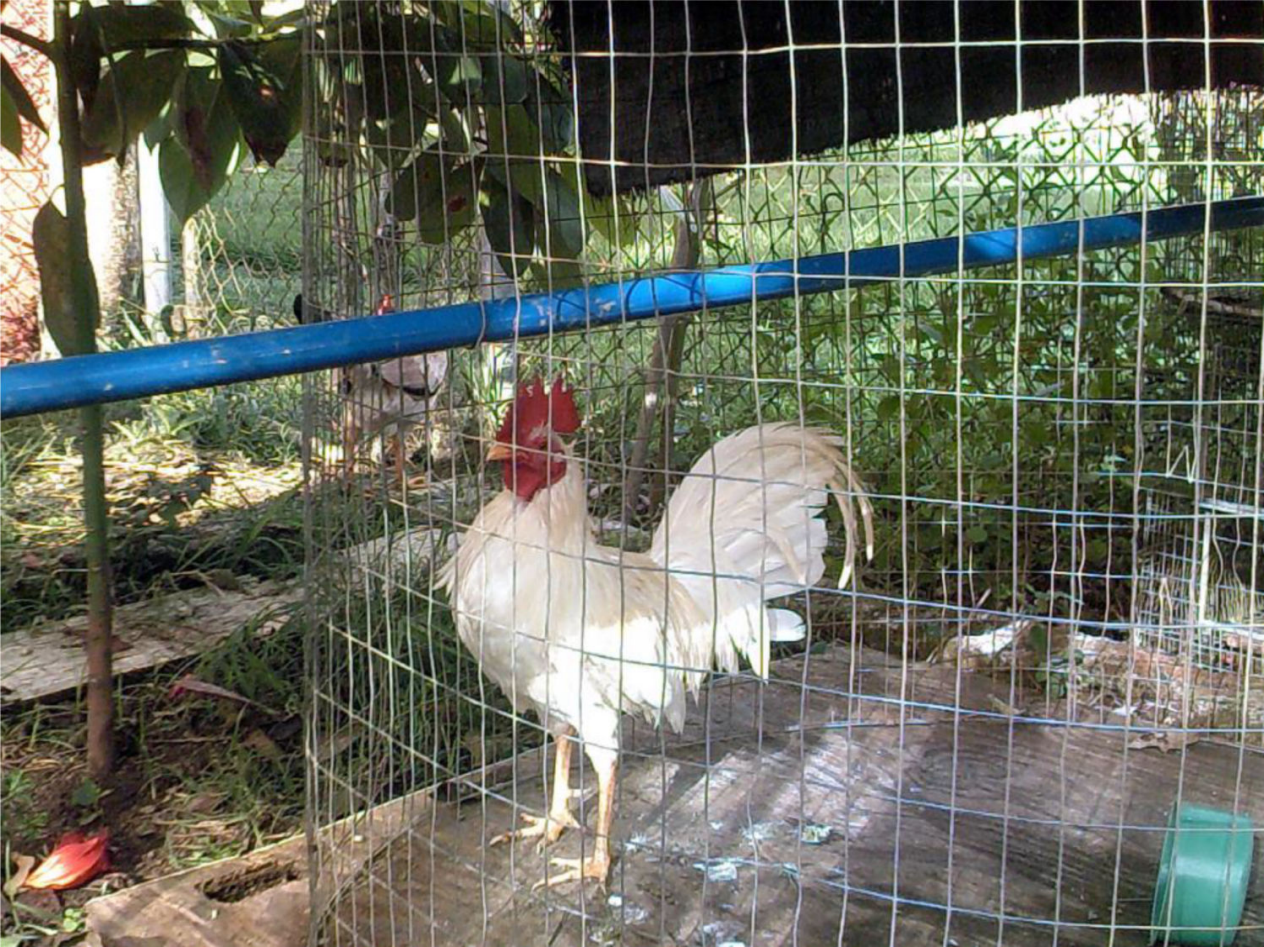
Rooster

**Figure 8.**
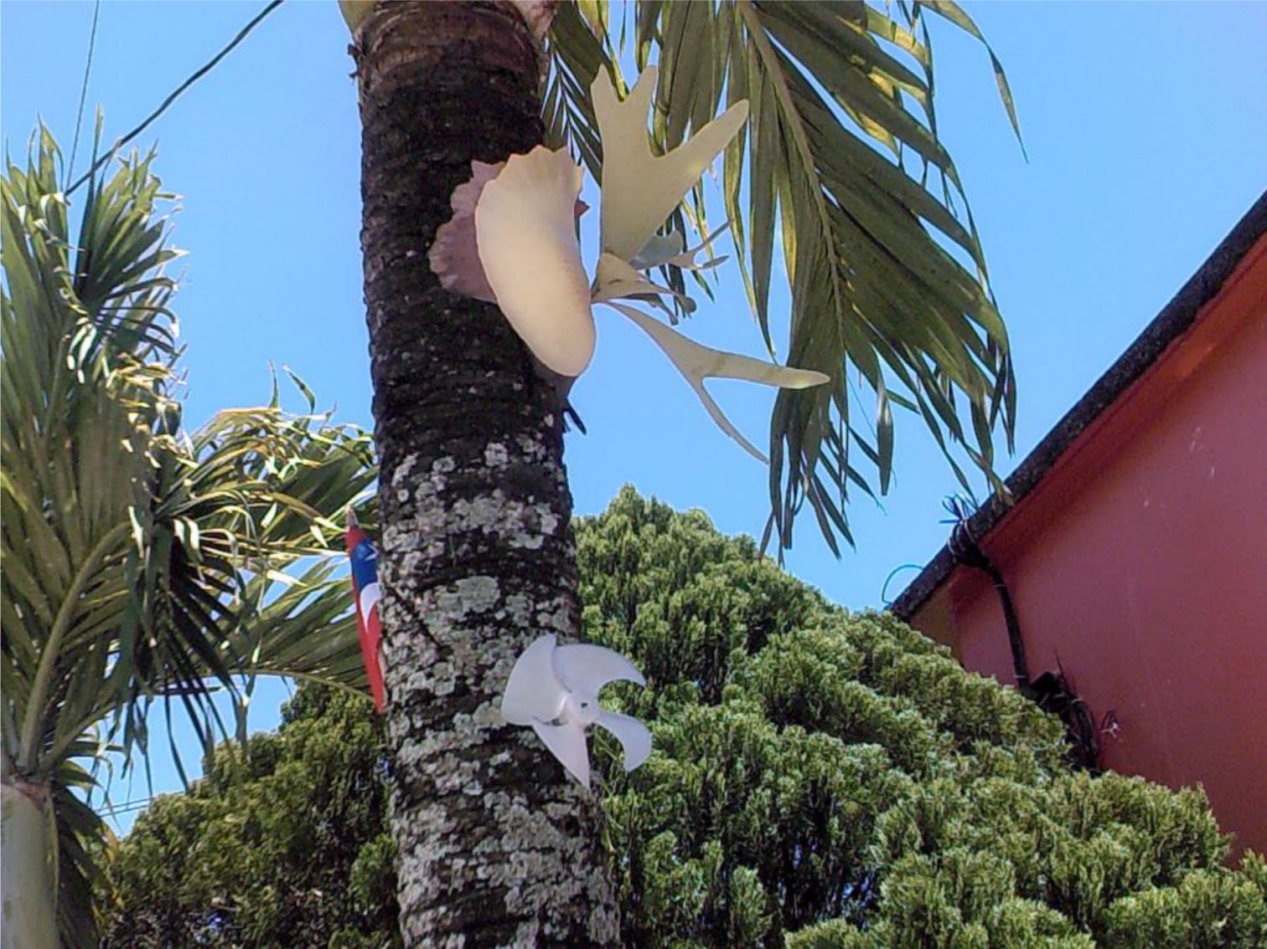
Front yard, with Puerto Rican flag on tree
